# Bulk and Surface Acoustic Wave Biosensors for Milk Analysis †

**DOI:** 10.3390/bios12080602

**Published:** 2022-08-05

**Authors:** Kerstin Länge

**Affiliations:** Institute of Microstructure Technology, Karlsruhe Institute of Technology, Hermann-von-Helmholtz-Platz 1, 76344 Eggenstein-Leopoldshafen, Germany; kerstin.laenge@kit.edu

**Keywords:** bulk acoustic wave, surface acoustic wave, quartz crystal microbalance, EMPAS, milk, proteins, toxins, drug residues, pathogens, lab-on-a-chip

## Abstract

Milk and dairy products are common foods and, therefore, are subject to regular controls. Such controls cover both the identification and quantification of specific components and the determination of physical parameters. Components include the usual milk ingredients, mainly carbohydrates, proteins, and fat, and any impurities that may be present. The latter range from small molecules, such as drug residues, to large molecules, e.g., protein-based toxins, to pathogenic microorganisms. Physical parameters of interest include viscosity as an indicator of milk gelation. Bulk and surface acoustic wave sensors, such as quartz crystal microbalance (QCM) and surface acoustic wave (SAW) devices, can principally be used for both types of analysis, with the actual application mainly depending on the device coating and the test format. This review summarizes the achievements of acoustic sensor devices used for milk analysis applications, including the determination of physical liquid parameters and the detection of low- and high-molecular-weight analytes and microorganisms. It is shown how the various requirements resulting from the respective analytes and the complex sample matrix are addressed, and to what extent the analytical demands, e.g., with regard to legal limits, are met.

## 1. Introduction

Milk and dairy products, particularly those derived from cows, are part of many people’s diets. Milk meets several needs due to its diverse composition. The main components are water, carbohydrates, proteins, and fat, followed by minor components, such as mineral salts and vitamins. However, milk may also contain harmful contaminants, for instance, pathogenic microorganisms and their toxins, drug residues remaining from veterinary treatments, and pesticides that have been ingested through feed but are not fully metabolized. Furthermore, since milk is a polydisperse system, milk quality is also related to its physical and mechanical properties. Milk density, for instance, can indicate improper dilution with water, while the viscosity depends on the age and composition of the milk. Furthermore, the viscosity is affected by mechanical and thermal treatments to which the milk was subjected. Consequently, a comprehensive analysis of milk samples requires both the specific detection of milk components, be it natural ingredients or contaminants, and the determination of the physical parameters of the milk as a whole [1,2,3,4].

A large variety of standard procedures is already available for food analysis and, hence, milk analysis. Atomic absorption spectroscopy (AAS) and atomic emission spectroscopy (AES) are applied for the determination of major and trace elements. Ultrasound spectrometric methods and optical spectroscopic methods, such as ultraviolet/visible (UV/VIS), VIS/near-, and middle-infrared (VIS/NIR and VIS/MIR) spectroscopy, are used for quantitative and qualitative milk analysis. These techniques are sometimes incorporated into milk processing equipment or milking systems for real-time monitoring of the milk parameters. The latter often includes measuring cells for determining the conductivity via the measured impedance since an increased conductivity may be an indicator of bovine mastitis. Furthermore, chromatographic separation combined with subsequent detection are applied for the detection of milk components, such as gas chromatography (GC) or high-performance liquid chromatography (HPLC) coupled with mass spectrometry (MS) or UV/VIS spectroscopy. HPLC can especially be used to separate and identify almost any compound that may be present in milk since several stationary and mobile phases are available. Finally, immunological methods are used for the detection of food allergens, other proteins, and microorganisms. These methods include immunoprecipitation techniques, such as immunodiffusion and agglutination, enzyme-linked immunosorbent assay (ELISA) test formats, and lateral-flow assays (LFAs). LFAs are mainly being used for sample screening or field tests since they are associated with the least experimental effort but may lack sufficient accuracy. In addition, the polymerase chain reaction (PCR) is applied to identify and detect bacterial DNA in milk [1,2,3,4,5,6].

Integrated receptor-transducer devices, i.e., biosensors, are principally suitable for both specific analyte detection and, when focusing on the transducer part, for measuring physical parameters. Setups allowing label-free detection are particularly advantageous since they enable fast and specific determination of analyte concentrations with low experimental effort. However, in both types of analytical problems, the complexity of the sample matrix has to be considered when developing the respective device coating since non-specific binding to the biosensor or the transducer surface would interfere with the results. Nevertheless, biosensor setups have successfully been used for a variety of sensing applications in milk. Examples include the investigation of liquid properties and the detection of bacteria, proteins, drugs, hormones, pesticides, and mycotoxins, i.e., the scope of analytical tasks required for milk samples can be covered by biosensors [7,8,9,10,11,12,13,14]. Biosensor detectors typically use electrochemical, optical, or acoustic signal transduction. While electrochemical biosensors are the ones most commonly used, optical and particularly acoustic biosensors offer higher flexibility regarding user-defined coatings and sensing layers that do not interfere with the transduction principle. Another advantage of acoustic biosensors is that mass loading has a major effect on the sensor signal. Since mass is an inherent property of any analyte, this makes those sensors universal in use. Because of the impact of mass on the sensor response, acoustic biosensors are also classified as gravimetric (mass-sensitive) biosensors. Furthermore, they are also classified as piezoelectric biosensors because of their operating principle (see Section 2.1). The best-known representatives of acoustic biosensor transducers are quartz crystal microbalances (QCMs) and surface acoustic wave (SAW) devices [15,16,17,18,19,20].

QCM sensors have their origin in quartz crystal resonators developed about a century ago to control the frequencies in oscillators [21,22]. The development of SAW devices for high-frequency technologies was stimulated in the mid-1960s by the introduction of planar interdigital transducers (IDTs), which allow for the generation and detection of SAWs on the crystal surface (see Section 2.1) [23,24]. Both quartz crystal resonators and SAW devices continue to be important parts in electronic applications today, such as resonators for oscillators, filter elements, and other components for use in data processing and telecommunications. This makes the devices universally available or at least allows for the comparatively simple and inexpensive production of customized designs since the manufacturing processes are established [18,22,25]. The first applications of quartz crystal resonators as microbalances, which were reported in the late 1950s, were mainly focused on thickness determination of metal films, for which QCMs are still used today, e.g., during the vapor deposition process [26,27]. This was quickly followed in the early 1960s by the first gas-sensing experiments with selectively coated QCM devices, which was followed a decade later by the first QCM biosensor measurements using a biospecific layer [28,29,30]. The corresponding experiments with SAW sensor devices did not follow until the late 1970s and thereafter owing to the fact that the concept of IDTs was not introduced until the mid-1960s, as mentioned before [23,24,25,31]. Today, both QCM and SAW gas sensors and biosensors have successfully been applied in several analytical applications, covering the areas of medical diagnostics, food safety, and environmental analysis [18,19,27]. Nevertheless, acoustic biosensors are often underrepresented compared with biosensors based on electrochemical and optical detection. Therefore, to come back to the topic of milk analysis mentioned above, this review specifically considers acoustic biosensor applications in the analysis of milk to demonstrate their performance in this field. In the following, the basics of QCM and SAW devices for liquid sample measurements are described, followed by considerations regarding the experimental requirements for analytical measurements in the complex matrix of milk. After that, achievements obtained with acoustic biosensor setups in milk analysis are summarized, including the determination of physical parameters and the specific detection of a wide variety of analytes. Finally, the acoustic biosensor performance capabilities are compared with the standard methods of the corresponding applications to allow for an estimation of the future perspective of acoustic biosensors.

## 2. Acoustic Wave Biosensors for Measurements in Milk Samples

### 2.1. Bulk and Surface Acoustic Wave Sensor Devices for Use in Liquid Applications

**Figure 1:**
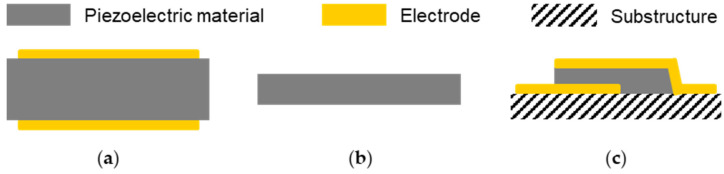
Schematics of bulk acoustic wave devices: (**a**) quartz crystal microbalance (QCM); (**b**) electromagnetic piezoelectric sensor (EMPAS); (**c**) film bulk acoustic resonator (FBAR).

The operation principle of bulk and surface acoustic wave sensor devices exploits the piezoelectric and the inverse piezoelectric effect. The piezoelectric effect describes the appearance of electrical charges on the surface of certain materials, such as crystals without inversion symmetry, by applying a mechanical force that deforms these materials. The deformation causes a displacement of the positive and negative charges and, hence, the charge centers, which no longer neutralize each other due to the asymmetry, resulting in the surface charges. This effect is reversible, which is then called the inverse (or reverse) piezoelectric effect. Here, an electric field applied to a piezoelectric material leads to mechanical distortion. Consequently, applying an alternating electrical field results in alternating distortions, i.e., a mechanical (acoustic) wave. By using the interconversion and detection of electrical energies and acoustic waves, acoustic sensors and biosensors enable label-free, fast, sensitive, and low-cost detection of analytes in both gaseous and liquid samples. However, applications in liquids require particle displacements that are parallel to the device surface; otherwise, the insertion loss would be too high. This is fulfilled, for instance, by QCMs, which are the oldest and still most commonly used acoustic sensor devices; furthermore, it is beneficial that QCM-based biosensor instruments are commercially available. QCMs support thickness shear modes (TSMs) and, therefore, belong to the bulk acoustic wave (BAW) devices (Figure 1). The typical setup consists of a quartz (SiO_2_) disk, usually made of AT-cut quartz, with electrodes mounted on both surfaces (Figure 1a). The main parameter recorded during the measurements is the resonance frequency, which is linked to the deposited mass but also related to viscosity changes near the sensor. Furthermore, some instruments allow the additional recording of the dissipation of the signal to gain insight into viscoelasticity changes within deposited and coating layers resulting from binding processes [18,19,27,32,33].

Common QCM frequencies are in the range of 5 to 50 MHz. Higher device frequencies would be desirable since this would promise higher sensitivities to mass loading. However, this is difficult to realize with QCM sensors since higher frequencies would result in thinner devices, making them fragile and difficult to handle, including the fluidic and electrical contacting. Recent developments to obtain higher BAW frequencies led to electromagnetic piezoelectric sensors (EMPAS) (Figure 1b) and film bulk acoustic resonators (FBARs) (Figure 1c). An EMPAS uses a thin AT-cut quartz crystal that is placed near a planar electromagnetic copper coil. Mechanical oscillations on the quartz are caused by an electric field, which is generated by an electromagnetic field resulting from the current flow in the coil. EMPAS measurement frequencies are odd multiples of the fundamental frequency, i.e., a fundamental frequency of 20 MHz, for instance, allows for operating frequencies up to 1 GHz. On the other hand, FBARs are mostly made of aluminum nitride or zinc oxide thin films that are mounted on a support structure. The resonators can be operated in a longitudinal mode or a TSM at device frequencies from sub-GHz to tens of GHz. However, in liquids, a TSM is preferred to minimize energy loss. Both EMPAS and FBARs have been applied in biosensing applications [18,19,34,35,36].

**Figure 2:**
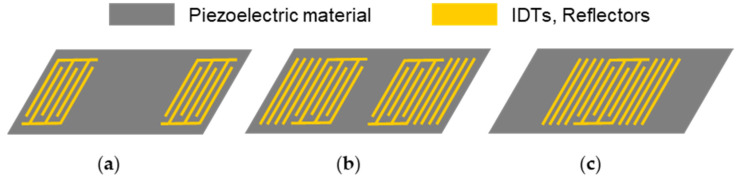
Schematics of surface acoustic wave devices: (**a**) delay line configuration; (**b**) two-port resonator; (**c**) one-port resonator.

Though less common than QCM sensors, SAW sensors represent the other large group of acoustic sensors. The acoustic wave propagates on the surface of the piezoelectric substrate, where it is excited and received by IDTs. The layout of the IDTs mainly follows two designs, i.e., a delay line or a resonator configuration. In the delay line configuration (Figure 2a), the spacing between input and output IDTs causes a time delay between the respective signals. The resulting SAW is usually recorded by following phase and amplitude shifts, which require comparatively complex electronic setups. SAW resonators possess additional reflective fingers surrounding the IDTs, which are closer together than in the delay line configuration (two-port resonator, Figure 2b) or reduced to one IDT (one-port resonator, Figure 2c). The distinct and sharp resonance frequencies that result from the resonator configurations can easily be collected by simple and economical electronic setups, such as oscillators [20,25,32].

As mentioned before, particle displacements parallel to the device surface are required for applications in liquid samples, leading to SAW devices operating with shear horizontal particle displacements, such as horizontally polarized shear waves. This is supported by specifically cut piezoelectric substrates, e.g., ST-cut quartz, 64°YX-LiNbO_3_, and 36°YX-LiTaO_3._ These substrates are often combined with a waveguiding structure to confine the wave at the surface by hindering wave components from radiating into the bulk substrate. Wave guidance may be obtained by thin guiding layers, resulting in Love waves, or by metal strip gratings, leading to surface transverse waves [20,25,37,38].

### 2.2. Measuring with BAW and SAW Sensors

The crystal cut of acoustic sensor devices is primarily chosen according to the application medium; however, if possible, the temperature stability of the piezoelectric materials should also be considered. While AT-cut quartz, as is typically used for QCM devices, provides comparatively stable frequencies over a wide temperature range, materials used for SAW devices, such as LiNbO_3_ and LiTaO_3_, show higher frequency changes associated with temperature changes. Additional quartz layers on the respective SAW devices can reduce these effects, but a more suitable measure may be the integration of an external thermostatic control in the final measurement setup since this would also allow for the temperature control of any solution involved in the measurement [18,19,32,37].

Considering mass loading as the main effect on the device frequency, high device frequencies are desirable since they would lead to larger frequency shifts. However, a higher mass sensitivity is only achieved if the higher operating frequencies are not associated with an equally higher noise [18,37]. Furthermore, higher device frequencies are also linked with reduced penetration depths, i.e., the sensing zone of the acoustic wave into the bulk of the medium, including the sensing layer, is limited. Another effect on the biosensor response results from the composition of the sensing layer with regard to viscoelasticity, which is also associated with the penetration depth of the acoustic wave. The effects of mass loading and viscoelasticity change on the sensor signal may add to each other, but they may also counteract each other, leading to reduced sensor responses. Disadvantageous effects that result from reduced penetration depth or counteracting viscoelasticity changes can be minimized by the use of thin, two-dimensional sensing layers, which enable analyte binding mostly on top of the layer. Consequently, high-performance acoustic biosensor setups require the adaptation of both the sensor device and the device coating to the respective application [18,19,39,40,41,42].

Apart from binding events on the device surface, acoustic biosensor signals also respond to differences in the physical liquid parameters of the sample liquids. Such differences may already occur when switching between a carrier medium transporting a sample to the sensor and the sample itself. This effect was successfully exploited for density and viscosity measurements [14,43,44,45,46]. On the other hand, changes in the electrical environment may also influence the electromechanical coupling of the acoustic device and, hence, have a high impact on the biosensor signal response. This problem has effectively been eliminated from QCM and SAW delay line sensors by introducing metal coatings, which are able to shield the acoustic wave from differences in the electrical parameters, such as conductivity. However, such layers are difficult to implement on SAW resonators, mostly for steric reasons. Adapting the carrier medium to the sample matrix in a way that electrical differences are minimized offers a possibility to overcome this problem. A more recent approach combined SAW resonators with electrical sensors to obtain a dual-signal response, allowing for improved characterization of the individual sensor signals [14,19,43,47,48,49].

### 2.3. Testing Milk Samples with BAW and SAW Biosensors

#### 2.3.1. Capture Molecules

Similar to other bioanalytical assays, specific analyte detection with acoustic biosensors requires analyte-specific capture molecules or at least highly selective recognition structures. As shown in Section 3, bulk and surface acoustic wave biosensors mainly use antibodies, aptamers, and molecularly imprinted polymers (MIPs) as biorecognition elements for analyte detection in milk samples.

Antibodies belong to the most commonly used capture molecules since they can be developed for a large variety of corresponding antigens, i.e., target analytes, with very high specificity and affinity. However, since antibodies are proteins, they may lack stability, particularly if regeneration of the immunosensor coatings is required. In contrast to this, oligonucleotides are very stable but are restricted to the corresponding DNA or RNA strands as an analytical target. This led to the development of aptamers, i.e., oligonucleotides with defined structures. Aptamers are stable like regular oligonucleotides and they can be designed to bind to almost any target analyte with high specificity and affinity, similar to antibodies. Furthermore, MIP layers have been developed, which offer highly selective binding with high binding capacity, particularly for small analyte molecules,. A more recent approach was the combination of MIPs with aptamers to increase the selectivity while maintaining stability [50,51,52,53,54,55,56,57].

#### 2.3.2. Biosensor Test Formats

**Figure 3:**
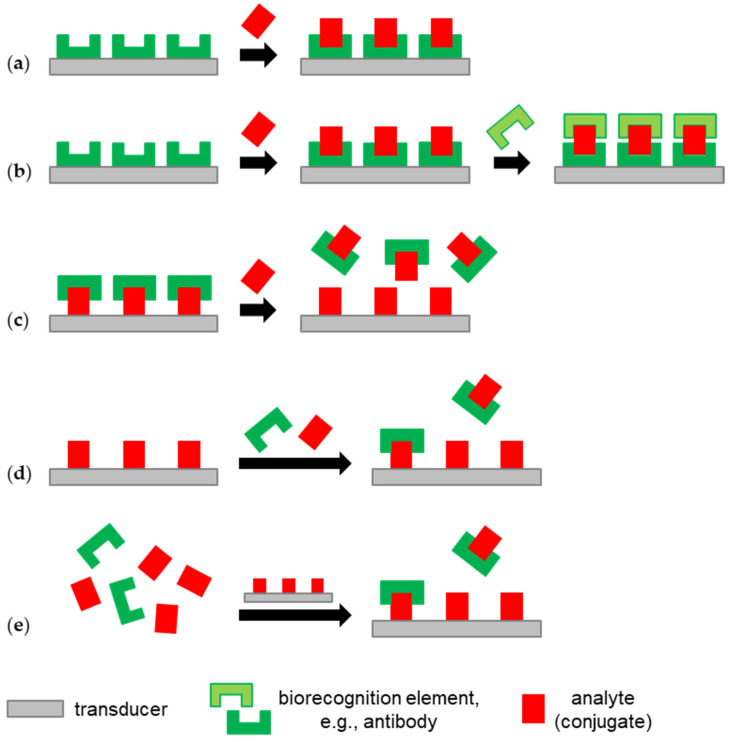
Label-free biosensor test formats used for analyte detection in milk with acoustic biosensors (see Section 3): (**a**) direct detection; (**b**) sandwich assay; (**c**) displacement assay; (**d**) competitive assay; (**e**) binding inhibition assay.

Bulk and surface acoustic wave biosensors allow for label-free analyte detection, for which various test formats are available. The fastest and easiest way to detect the target analyte is direct detection, i.e., the analyte binding on the biosensor surface coated with analyte-specific capture molecules results in a signal response (Figure 3a). However, a significant signal response requires a certain size and amount of analyte binding on the surface. Providing a sufficient size of the analyte, the signal response can be enhanced by another capture molecule binding to the analyte captured on the surface, which leads to the so-called sandwich assay test format (Figure 3b). Labeling the other capture molecule, e.g., using nanoparticles, may further enhance the signal response [15,58].

A variation of the direct detection assay is the displacement assay, which takes mass away from the surface instead of adding it. This is performed, for instance, by providing a biosensor surface with an immobilized analyte and capture molecules that bind to this analyte. If an analyte sample is added, capture molecules move away from the immobilized analyte and bind to the analyte in the sample solution instead (Figure 3c) [58].

The challenge in the determination of low concentrations of small, i.e., low-molecular-weight, analytes with acoustic biosensors is that such molecules cause only a small signal response. Signal enhancement using a sandwich assay is hindered by steric reasons. If this cannot be solved by developing surfaces with a suitably high binding capacity (e.g., MIPs, see above) or by enrichment measures (see Section 3.5), competitive (Figure 3d) and binding inhibition (Figure 3e) assays offer solutions here. Both assay types use samples that are mixed with a known concentration of the capture molecule, which is typically an antibody, and biosensor surfaces coated with an analyte. Analyte immobilization may be obtained by using an analyte derivative or an analyte–protein conjugate. The latter is typically called a hapten–protein conjugate, where “hapten” refers to a molecule that is too small to trigger an immune response on its own. In the competitive assay (Figure 3d), an analyte in the sample and an analyte immobilized on the surface compete for the binding sites of the antibodies added to the sample. In the binding inhibition assay (Figure 3e), the equilibrium of the binding reaction of an analyte and the added antibodies in the sample is awaited before the mixture is applied to the analyte-functionalized biosensor, where the remaining antibodies with free binding sites will bind [15,58,59,60].

Sandwich, competitive, and binding inhibition assays, where the capture molecules are typically antibodies, are also available as indirect test formats. In this case, additional antibodies (optionally labeled) bind specifically to the antibodies that are already binding via analyte (derivative) on the surface. This allows for the enhancement of both the signal response and the specificity of the respective assay [60].

#### 2.3.3. Dealing with the Milk Sample Matrix

Milk is a complex polydisperse system that consists mainly of water, carbohydrates, proteins, and fat. Particularly proteins and fat tend to adhere non-specifically on surfaces, which typically interferes with the results obtained with label-free biosensor setups. Non-specific protein adsorption from the sample may be hindered by using blocking solutions prior to the measurement. Such solutions contain surfactants, proteins, or even an analyte-free matrix (here: milk) to occupy the “non-specific binding sites” prior to the application of the real sample. However, the blocking agents themselves might interact with the sample components, and the binding efficiency of the surface might be reduced. Therefore, several coatings have been developed for use as intermediary biosensor layers, which could effectively reduce unwanted matrix effects. Most of these coatings use hydrogels, such as dextrans or polyethylene glycols (PEGs). Recent developments for acoustic biosensors have aimed at thin layers, such as PEGs with shorter chain lengths, resulting in oligo- or even monoethylene-glycolated coatings. However, even if the irreversible, non-specific protein adsorption is minimized, particularly the fat may still interfere with the assay reaction [13,15,61,62,63].

Interferences from the milk matrix can also be reduced via sample pretreatment methods. Several procedures have been introduced and adapted according to the type of target analyte, i.e., low- or high-molecular-weight compound or microorganism. The easiest pretreatment method is sample dilution, e.g., with a buffer. However, since this would also reduce the concentration of potential contaminants, mere sample dilution can only be used effectively for large concentrations of target analytes. Centrifugation is used to separate the fat, which can be skimmed off as the top layer after this treatment. Furthermore, a protein pellet consisting mostly of caseins may be collectible at the bottom of the centrifuge vial. Protein separation is further promoted by mixing the milk with ammonium sulfate, an organic solvent, or an acid since these additives lead to the precipitation of milk proteins so that they can easily be removed by subsequent centrifugation or filtration [1,12,64,65,66,67].

## 3. Application of BAW and SAW Sensors and Biosensors in Milk Measurements

The QCM devices mentioned in the following are made of AT-cut quartz and support frequencies in the range of 5 MHz to 20 MHz. Details of other sensors are described in the corresponding sections. Milk samples are typically delivered to the sensor by using flow systems and flow cells or by pipetting into stopped-flow setups, with measurements being performed under flow or static conditions, respectively. Unless otherwise noted, signal responses were directly taken from the liquid samples, as is common practice. However, in a few cases, the sensors were rinsed and dried before the signal readout; this is pointed out in the relevant sections. The limit of detection (LOD) data listed in the following include both experimentally determined and theoretically approximated values, depending on the information given in the related literature.

### 3.1. Coagulation Monitoring—Measurements of Physical Liquid Parameters

QCM sensors were utilized to monitor milk gelation caused by acidification or by adding rennet. The quartz substrates had no surface coating other than the gold electrodes, with one electrode per QCM being in contact with the sample. Since mechanical (acoustic) and electrical parameters are linked by the piezoelectric effect, electrical impedance measurements were performed instead of frequency measurements to follow changes in the TSM resulting from changes in mass and rheological parameters. This setup allowed for the monitoring of the pH-dependent formation of casein clusters and milk gel networks. Furthermore, it allowed for the evaluation of the impact of temperature and rennet type on the gelation of the milk [68,69].

A commercially available viscosity sensor based on a BAW device is the ViSmart^TM^ viscosity sensor from Vectron International (headquarters: Hudson, NH, USA). The sensor utilizes langasite (La_3_Ga_5_SiO_14_ (LGS)), which shows a higher piezoelectric coupling than quartz. TSMs comparable to those obtained with AT-cut quartz are achieved with Y-cut LGS, i.e., the setup of the BAW device is similar to that described before (Section 2.1), with one electrode being in contact with the sample. This electrode is coated with diamond-like carbon for better chemical resistance since the intended use of the sensor is the viscosity determination of oils, lubricants, fuels, resins, and inks. Viscosity values are given in acoustic viscosity (AV) units, with the acoustic viscosity being the product of the dynamic viscosity and density of the liquid [70,71,72,73].

The ViSmart^TM^ viscosity sensor is not qualified for use in foods [70]; nevertheless, it was introduced for measurements in milk. The acoustic viscosity of reconstituted skim milk samples with total solid (TS) concentrations ranging from 10% to 40% was measured under static and flow conditions. Shear-thinning effects resulting from the non-Newtonian behavior of higher concentrated samples (TS > 30%) in the flow could be observed with both the ViSmart^TM^ viscosity sensor and reference measurements of the dynamic viscosity with a rheometer. A non-linear regression model was derived for the relationship between associated acoustic and dynamic viscosity values of the milk samples, where any potential mass adsorption resulting from the sample matrix was not considered further [74]. Furthermore, the ViSmart^TM^ viscosity sensor was applied to monitor acid-induced milk gelation at different acidulant concentrations and temperatures. The respective gelation points defined by reference rheometer measurements could also be specified with the viscosity sensor and a newly defined acoustic viscosity parameter. Mass effects resulting from the sample matrix were considered to be low because of the low operating frequency of the sensor (5.3 MHz) [75].

SAW resonators (36°YX-LiTaO_3_, 426.4 MHz) for use with low-fat UHT milk (1.5% fat) were coated with hydrogel to suppress mass adsorption from the sample matrix. The coating was efficient enough to reduce the non-specific adsorption of milk proteins to a minimum so that the remaining signal response was affected only by changes in the physical liquid parameters. However, since SAW resonators respond to both mechanical and electrical variations in the fluidic environment, suitable models that utilize reference measurements are still to be developed [76].

### 3.2. Determination of Fat Content

As described in Section 2.3.3, milk fat may interfere with the acoustic sensor measurements, which is why the fat is typically removed. On the other hand, fat itself was separated for determination with acoustic sensors. Fat extraction was performed with an organic solvent mixture or via supercritical fluid extraction and an uncoated QCM sensor with gold electrodes was used for detection. The results obtained with whole milk, low fat, or skim milk samples containing between 1% and 25% fat were similar to those obtained with reference fat determination methods, suggesting the QCM method as an economic screening tool for fat content in milk [77,78].

Furthermore, a shear horizontal SAW dual-delay-line device (36°YX LiTaO_3_, 61.18 MHz) was used to distinguish between milk types with different fat contents. One delay line was electrically shielded by a metalized layer to measure the mechanical properties of the liquid by responding to mass loading, viscosity, and density changes. The other delay line was left uncoated to respond additionally to the liquid’s relative permittivity and conductivity. Whole milk (4% fat), semi-skimmed milk (2% fat), and skimmed milk (no fat) could be distinguished well. Additionally, measurements with diluted whole milk samples resulted in a limit of detection of approximately 0.1% fat content [14].

### 3.3. Detection of Proteins in Milk

Proteins are usually high-molecular-weight compounds; therefore, they can principally be detected directly using acoustic biosensors without additional measures. Table 1 lists the achievements of QCM sensors applied for protein detection in milk. The proteins had in common the fact that the relative molecular mass was above 10,000 in each case. Proteins in milk are part of the normal ingredients but proteins may also be contained as elements of adulteration and contamination. Apart from selective and specific protein detection, QCM sensors were used for testing a model surface for monitoring food processing, taking advantage of the versatility of coating options for acoustic sensor devices. The coating used here was SS2343, which is a stainless-steel equivalent to 316 L. Raw milk used as whole or processed into different fractions was applied on the sensor surfaces at different temperatures, resulting in characteristic protein adsorption behaviors. The respective response curves were attributed to the initial layers of biofouling, which would allow for future investigations of cleaning operations for milk-processing units [65].

QCM sensors coated with aminated titanium dioxide were used for the detection of phosphoproteins. Phosphoproteins include caseins, which comprise about 80% of the milk proteins [1]. The selectivity of the binding was confirmed by subsequent elution of the proteins from the sensors and characterization by matrix-assisted laser desorption time-of-flight (MALDI-ToF) MS, leading to a good correlation between the QCM sensor and MALDI-ToF MS results obtained with diluted non-fat milk [79]. The protease plasmin, which may be associated with the casein micelles, is a minor component in milk, but may still affect the quality of the milk and the resulting dairy products. Plasmin was detected by exploiting its proteolytic function to cleave β-casein, where the use of the complete protein as a QCM biosensor coating was more successful than the use of a cleavable peptide. In contrast to an antibody coating, which would lead to the detection of the complete plasmin concentration, the use of the enzyme’s substrate as a sensor coating allows for the specific detection of the active plasmin [80,81]. Immunoglobulin G (IgG) is another minor component in milk. Increased concentrations indicate adulteration with colostrum, which may interfere with the milk quality, particularly if the colostrum concentrations are above 5%. Colostrum concentrations of only 0.1% to 2% led to IgG concentrations of 569 µg/mL to 1675 µg/mL in milk. After sample dilution by a factor of 1000, IgG was determined using QCM immunosensors, which provided a good correlation with the reference method, i.e., radial immunodiffusion [12].

Staphylococcal enterotoxins A and B (SEA and SEB) belong to a group of heat-resistant toxins, of which 100 ng are sufficient to cause food poisoning symptoms in an adult. Comparatively high SEB concentrations, i.e., 2.5 µg/mL to 10 µg/mL, were used for detection in fresh, low-fat, and skimmed milk samples, which were applied on QCM immunosensors without specific sample pretreatment [85]. Reducing the sample matrix to skim milk only enabled SEA detection with QCM immunosensors at concentrations below 1 µg/mL [82]. Further reduction of the sample matrix via protein precipitation allowed for QCM immunodetection of a few ng/mL SEA in spiked samples [83]. Similar results were obtained with MIP-coated QCM sensors for the detection of SEA and SEB [84]. Hence, reducing the complexity of the sample matrix principally favors the detection of smaller toxin concentrations in spiked samples. However, it has to be considered for future applications whether the sample pretreatment might affect real sample concentrations since protein-based toxins might be susceptible, for instance, to protein precipitation measures.

### 3.4. Detection of Low-Molecular-Weight Compounds in Milk

The low-molecular-weight compounds detected in the following had relative molecular masses below 500. Acoustic biosensor test formats included direct detection (Table 2), competitive, and binding inhibition assays (Table 3).

The low-molecular-weight analytes summarized in Table 2 and Table 3 cover both a dietary supplement (folic acid) and a wide range of contaminants, such as the adulterant melamine, an insecticide (endosulfan), mycotoxins (aflatoxin B1 and zearalenone), and drug residues. The latter includes a synthetic estrogen (diethylstilbestrol), thyroid medication (methimazole), and several antibiotics, such as the broad-spectrum antibiotic chloramphenicol, the aminoglycoside antibiotic tobramycin, β-lactam antibiotics (ampicillin and penicillin G), and fluoroquinolones (ciprofloxacin, enrofloxacin, levoflocacin). Some of the drugs are not approved for veterinary use in cows, though their administration may have been allowed in the past. Therefore, the current maximum residue limits (MRLs) valid in milk (MRL^milk^) are not available for all of these compounds. However, these drugs may still be applied illegally, such as methimazole or diethylstilbestrol as a means of animal fattening [93,98]. Hence, biosensor performances for those compounds may still be of interest. The results listed in Table 2 and Table 3 are mostly given as recovery values obtained with spiked milk samples, assuming that the used milk does not already contain the respective contaminants. This can usually be expected in the case of commercially available milk and is, therefore, sufficient for the evaluation of sensor coatings and for a first approximation of the biosensor efficiencies.

Most of the procedures for preparing milk samples for the detection of low-molecular-weight compounds (see Table 2 and Table 3) use means to remove fat and proteins, such as the addition of additives for protein precipitation, followed by centrifugation or filtration steps (see Section 2.3.3). This implies that the small analytes will be found in the supernatant or filtrate collected afterward. While this works in the majority of applications, possible exceptions should be taken into account. Such an exception is melamine, which could be detected in milk by direct detection via MIPs. However, melamine detection in milk was only successful if the melamine was added to the milk after sample pretreatment, i.e., after the milk had been diluted or after protein precipitation and centrifugation of the milk samples. Otherwise, melamine would interact with the milk proteins such that it is no longer accessible after protein precipitation, which would make direct detection of this adulterant impossible [91,92].

Direct detection of low-molecular-weight compounds with label-free biosensors is challenging, especially when dealing with low concentrations, because of the reduced mass loading. Therefore, QCM sensors for direct detection of such analytes (Table 2) used coatings with a high or increased binding capacity, such as MIPs (see Section 2.3.1), structured surfaces, or a combination of both, to enhance the potential mass load. Small concentrations were not in demand when it came to the detection of folic acid in baby milk formulations, where an MIP layer enhanced with metal-chelate for improved selectivity was exploited to detect defined contents of 150 ng/mL and 180 ng/mL folic acid [90]. Furthermore, MIP-coated QCM sensors were applied for the detection of sub-ng/mL concentrations of tobramycin [94] (MRL^milk^: n/a [101,102]), 1 ng/mL chloramphenicol [86] (MRL^milk^ 2003: 0.3 ng/g [86,96]; meanwhile prohibited [101,102]), and 10 ng/mL to 40 ng/mL enrofloxacin [89] (MRL^milk^: 100 ng/mL for enrofloxacin and ciprofloxacin together [89]). Furthermore, MIPs structured with microspheres and hollow (micro-)spheres of 400 nm to 500 nm diameter were used for the detection of 5 ng/mL to 100 ng/mL endosulfan [88] (MRL^milk^: 0.01 mg/kg ≈ 10 ng/mL [102]) and 50 ng/mL to 200 ng/mL methimazole [93] (MRL^milk^: n/a; prohibited [103]), respectively. Finally, multi-walled carbon nanotubes (MWCNTs) were used for structuring QCM immunosensor layers to detect 50 ng/mL to 300 ng/mL ciprofloxacin (MRL^milk^: 100 ng/mL for ciprofloxacin and enrofloxacin together [89]) or levofloxacin (MRL^milk^: n/a [101,102]) [87].

Competitive and binding inhibition assays avoid potential problems of reduced mass loads of low-molecular-weight analytes by instead detecting the corresponding capture molecules, which were specifically added to the samples (see Section 2.3.2). As was shown for the detection of ciprofloxacin and levofloxacin (MRL^milk^: see above) in buffer, the LOD values obtained with direct detection could be reduced by a competitive assay from 21 ng/mL and 25 ng/mL to 8 ng/mL and 9 ng/mL, respectively. Consequently, lower concentrations could be applied for detection in milk using the competitive assay, i.e., 20 ng/mL to 70 ng/mL instead of 50 ng/mL to 300 ng/mL, as is used for the direct detection assay. In both cases, MWCNTs were used for surface structuring. Whether the competitive assays were carried out under static conditions with sensor responses extracted after rinsing and drying or under fluidic conditions with the signal readout taken from the liquid samples did not greatly influence the LOD values in the buffer [87].

Generally, competitive and binding inhibition assays with acoustic sensors coated with hapten (Table 3) were carried out with smaller concentrations than applied for direct detection (Table 2). Competitive assays with QCM sensors were performed for the detection of 5 ng/mL and 10 ng/mL chloramphenicol [96] (MRL^milk^: as mentioned above), 5 ng/mL to 100 ng/mL zearalenone [97] (MRL^milk^: n/a, since occurrence is mainly in cereals [97]), and 10 ng/mL or 20 ng/mL ampicillin and/or penicillin G [95] (MRL^milk^: 4 ng/mL each [95,101]). In the case of the latter, the recoveries were unusually high, with values starting at 126%, which was explained as an already present level of antibiotic in the original milk. Furthermore, milk samples containing 0 ng/mL to 10 ng/mL penicillin G were classified via binding inhibition assays with SAW resonators (36°YX LiTaO_3_, 428.5 MHz) according to the concentration being above or below the MRL in milk [13]. Labeled antibodies were applied to enhance the QCM sensor signal response in competitive assays and enable the detection of even lower concentrations. For instance, competitive assays for the detection of 0.5 ng/mL to 50 ng/mL diethylstilbestrol (MRL^milk^: n/a) were conducted using gold-labeled antibodies instead of the usual non-labeled antibodies [98]. Furthermore, indirect competitive assays were performed with labeled secondary antibodies for the detection of 0.1 ng/mL to 10 ng/mL aflatoxin B1 (MRL^milk^: 2 µg/kg ≈ 2 ng/mL). Labels were gold nanoparticles or horseradish peroxidase (HRP), with the latter catalyzing the hydrogen peroxide-induced oxidation of 4-chloro-1-naphthol to the insoluble benzo-4-chlorohexadienone [99,100].

In summary, most low-molecular-weight compounds can be detected with acoustic biosensors in the relevant concentration ranges, particularly when competitive test formats, optionally enhanced by the use of labeled antibodies, are applied.

### 3.5. Determination of Bacteria in Milk

The majority of the bacteria detected by acoustic biosensors are common foodborne pathogens, such as *Salmonella enterica* (*S. enterica*), *Listeria monocytogenes* (*L. monocytogenes*), *Escherichia coli* (*E. coli*), *Brucella melitensis* (*B. melitensis*), and *Bacillus cereus* (*B. cereus*). The detection of *Francisella tularensis* (*F. tularensis*) was also conducted, though its occurrence is less common in milk; however, since *F. tularensis* can be used as biological warfare agent, it cannot be ruled out completely. Furthermore, the use of acoustic biosensors for the detection of *Bifidobacterium bifidum* (*B. bifidum*) and *Lactobacillus acidophilus* (*L. acidophilus*) was investigated. These are probiotic bacteria, which may also be used in the production of fermented foods. While a certain quantity of probiotics is required for a positive outcome, the number of colony-forming units (CFU) of pathogens in milk should be minimal, particularly if the milk is to be consumed directly and not processed further since bacterial cells multiply excessively under the right conditions [104,105,106,107].

The following provides information regarding the direct detection, displacement, and sandwich assays that were conducted with acoustic biosensors to detect bacteria in milk (Table 4). Competitive and binding inhibition assays, which were useful for the detection of low-molecular-weight analytes (see Section 3.4), do not bring any advantages in the detection of bacteria. Instead, sample incubation after inoculation (i.e., spiking) was applied to obtain enhanced cell numbers and, thus, facilitate label-free detection. However, incubation times in the range of 2 h to 18 h (Table 4) led to accordingly longer assay times. Another means to increase the signal response obtainable by direct detection is the use of magnetic particles coated with capture molecules to collect the corresponding bacteria in the sample; the results are summarized in Table 5. Furthermore, cell lysis was applied and the released DNA was multiplied using PCR or loop-mediated isothermal amplification (LAMP) prior to acoustic sensor measurements (Table 6).

Sample pretreatment for bacteria detection in milk using direct detection, displacement, and sandwich assays was mainly limited to spiking and incubating. Dilution is typically included in the spiking step when samples are mixed with bacterial solutions. However, some assays applied additional dilution steps, which are mentioned in Table 4. Filtration and separation of the cells by centrifugation were each described only once, and these additional steps did not necessarily lead to the detection of smaller cell concentrations (Table 4).

Direct detection of the probiotics *B. bifidum* and *L. acidophilus* with antibody-coated QCM sensors resulted in a detection limit of 10^3^ CFU/mL in milk diluted by a factor of 100. The highest detectable cell concentration was 5 × 10^5^ CFU/mL [106]. A concentration within this measuring range, namely, 10^4^ CFU/mL *B. bifidum* in about 50× diluted milk, was tested using a sandwich assay with gold-labeled antibodies [114]. The dilution successfully reduced interferences from the milk proteins, but the LOD in undiluted milk was correspondingly higher. However, the lowest acceptable bacteria concentration for probiotic products could still be detected since they should at least contain a concentration of 10^6^ CFU/mL of probiotic bacteria [106].

Similar to the direct detection of probiotics, direct detection of pathogens in milk using QCM immunosensors was only possible at high concentrations, as shown with milk samples spiked with *E. coli* (10^7^ CFU/mL) [110], *F. tularensis* (10^5^ CFU/mL and 10^8^ CFU/mL) [111], and *S. enterica* (1.2 × 10^7^ CFU/mL to 4.8 × 10^7^ CFU/mL) [112]. An *E. coli* concentration of 10^2^ CFU/mL was only detectable after an 18 h incubation time when the concentration had grown to 10^6^ CFU/mL [110]. If only one pathogen is known to be present, the amount of that pathogen can also be determined without the use of capture molecules, as shown for the detection of *E. coli* with QCM sensors providing a gold electrode or a parylene C coating as a surface [108,109]. Pathogen concentrations detectable using direct detection with acoustic sensors are too high to allow for fast determination of relevant pathogen contents in milk, which is at most a few CFU/mL [104,107]. However, since these sensors allow for time-resolved monitoring of binding events on the surface, they can be used for monitoring bacterial growth, e.g., in fermenters, where low concentrations are typically not the limiting factor. This was demonstrated by monitoring *E. coli* cell proliferation with parylene-C-coated QCM sensors [109].

Using QCM sensors with test formats other than direct detection includes the sandwich assay mentioned above, where 10^4^ CFU/mL *B. bifidum* in about 50× diluted milk were detected and compared with the same concentration of non-specific bacteria [106]. Furthermore, a displacement assay for the detection of *L. monocytogenes* in milk was performed with approximately 10^6^ cells of each *Listeria* and non-specific *Serratia*, with the latter again used for comparison [113]. Though smaller concentrations could have been applied, these test formats did not improve the LODs in a way that a limit of a few CFU/mL could be detected with QCM biosensors. In contrast to this, when an aptamer-coated EMPAS device operated at 984 MHz was used for the direct detection of *E. coli*, an LOD of only 8 CFU/mL in milk was achieved [115].

Enhancing milk sample preparation using magnetic particle enrichment allows for the reduction of detectable bacteria concentrations in milk while still using direct detection with QCM (or SAW) biosensors. The essence of this strategy is that the bacteria in the milk sample are first collected by correspondingly coated magnetic particles and separated from the sample using an external magnet. This separates the bacteria from the complex sample matrix, which facilitates the subsequent biosensor measurement. Furthermore, bacteria may be concentrated by reducing the volume of the resuspension buffer compared to the volume of the original milk sample, and the particles themselves may be used to increase the mass load on the acoustic biosensor surface (Table 5).

Aptamer-coated magnetic particles were used to capture *B. melitensis* and *S. enterica*, which were subsequently eluted for detection with aptamer-coated QCM sensors. Concentrations of the corresponding bacteria in the order of 10^4^ CFU/mL resulted in significant frequency shifts, while similar concentrations of non-specific bacteria led to negligible or significantly reduced sensor responses [116,119]. Furthermore, an LOD of 10^3^ CFU/mL in milk was determined for *B. melitensis*. This concentration is still high for the application but it was lower than what was applied in the majority of particle-free direct detection assays performed with QCM sensors (Table 4) and the detection did not require several hours of sample incubation [116].

The detection of *L. monocytogenes* captured by antibody-modified magnetic particles was done without elution of the bacterial cells from the particles. The resulting mass load increase in the subsequent QCM immunosensor measurement led to an LOD in milk of three cells per 200 µL, corresponding to 15 cells/mL, while *Listeria* detection without the use of magnetic particles was not possible in this setup [118]. The mass load was further increased for the detection of *E. coli* by binding gold-labeled proteins on the magnetic particles, in addition to the captured bacterial cells. Furthermore, the gold labels were enhanced by catalytic growth. The subsequent QCM immunosensor measurement resulted in an LOD of 53 CFU/mL in milk [117]. Since *Listeria* and *Escherichia* were naturally detected with different antibodies, which may have different affinities to the respective target bacteria, and different frequency readouts were applied, i.e., in liquid and after drying, respectively, a direct comparison of the assay performances is not possible. However, both assays exploiting magnetic particles as additional means to increase mass loading showed LODs below 10^2^ cells/mL [117,118], which was lower than obtained by the assays working with eluted bacteria [116,119].

Another means to reduce the LOD values is cell lysis combined with PCR or LAMP for the amplification of the released DNA prior to detection with acoustic biosensors (Table 6). PCR amplification was used for the detection of *E. coli* in milk. Prior to amplification, the bacteria were separated from the pretreated milk sample via centrifugation. The target oligonucleotides obtained via PCR were detected using a QCM sensor coated with the corresponding oligonucleotides. This was followed by sandwich hybridization with gold-labeled oligonucleotides to enhance the mass load [120], similar to the sandwich assay described before (Table 4) for the detection of *B. bifidum* [114]. Measurements in buffer showed that the sandwich hybridization with gold labels allowed the reduction of the LOD for *E. coli* to 1.2 × 10^2^ CFU/mL, which is two orders of magnitude lower than that obtained using direct detection of the oligonucleotides. In milk, sandwich hybridization allowed for clear differentiation between samples spiked with 5.3 × 10^2^ CFU/mL and non-spiked samples [120].

Finally, LAMP was applied for the detection of *S. enterica* in milk. In this case, antibody-coated magnetic particles were used to separate the bacteria from the milk prior to amplification. The LAMP amplicons were detected with SAW delay line sensors (ST-cut quartz, 155 MHz) functionalized with poly(L-lysine). The complete procedure took only four hours and allowed for the detection of a single *Salmonella* cell contained in a 25 mL milk sample, provided the entire sample was processed [121]. The procedure was slightly revised to allow parts of it to be performed in a lab-on-a-chip platform developed specifically for this purpose. The main difference is that a centrifugation step is included to separate the bacteria pellet after a short incubation period from the milk, i.e., antibody-coated magnetic particles are not required anymore. The remaining processing of the pellet suspension, including bacteria capture, lysis, LAMP, and SAW sensor detection, was then carried out on the lab-on-a-chip system. It could be shown via spiking with *S. enterica*, *E. coli*, *B. cereus*, or *Listeria* that the detection of one to five cells in 25 mL milk is possible, meeting the limits of pathogen detection required for food safety [122].

### 3.6. BAW and SAW Biosensor Performance Compared with Standard Methods

As shown in the previous sections, acoustic biosensor setups were successfully used for a large number of applications in milk analysis. Regarding the specific detection of milk components and contaminants, direct detection with acoustic biosensors may offer a convenient alternative to more complex setups and operations, as they are found in combinations of chromatographic separation units with detection devices and in immunological methods, such as immunodiffusion and ELISA. However, to compete with the standard methods, reduced experimental effort is not enough; it is also important that the analytical tasks can be performed with similar performance.

No examples are listed above for the determination of major and trace elements in milk. It is principally possible to determine these components with acoustic sensors, e.g., using MIPs, chelating agents, or bacteria (e.g., *E. coli*) as recognition elements [123,124,125]. However, these sensors lack the required specificity and, therefore, cannot compete against the standard methods, i.e., AAS and AES [1]. Furthermore, the determination of physical liquid parameters, such as viscosity for coagulation monitoring, with acoustic biosensor transducers may be affected by mass adsorption (see Section 3.1). Though this can partly be avoided by appropriate surface coatings, the implementation of contactless methods, such as ultrasound spectrometric and optical spectroscopic methods, may be more suitable than the use of surface-sensitive transducers. In contrast to that, acoustic biosensor setups were suggested as economic screening tools for fat content in milk (see Section 3.2). In this case, the suitability would still have to be verified by means of comparative measurements with standard methods.

When it comes to high-molecular-weight compounds, such as proteins, large concentrations can easily be detected via direct detection with acoustic biosensors (see Section 3.3). Using IgG as an example, results obtained with the acoustic biosensor corresponded to those obtained by radial immunodiffusion used as a standard method but were achieved in only a few minutes instead of several hours [12]. However, since the significant IgG concentration in milk is rather high, the use of an LFA might be more suitable for a fast sample screening, depending on the required accuracy and the preferred measurement site. The detection of low protein concentrations in milk by ELISA allows for LOD values down to 0.25 ng/mL or even 0.05 ng/mL, depending on the sample preparation method, as shown by the example of staphylococcal enterotoxins [126]. The lowest LOD given in Table 1 is 0.4 ng/mL for the direct detection of SEA in a buffer by an acoustic immunosensor; however, this value was calculated from the calibration curve ranging from 1 ng/mL to 80 ng/mL [83]. Furthermore, the SEA or SEB concentrations applied in milk were at least 5 ng/mL (Table 1). Hence, in contrast to ELISAs, which generally promise low LODs [127], direct detection of small protein concentrations in milk with acoustic biosensors remains difficult. The use of acoustic biosensors with higher device frequencies and the application of other test formats, such as sandwich assays with optional labels for further signal amplification, could help to be able to use acoustic biosensors for this purpose in the future.

Similar to proteins, the direct detection of low-molecular-weight compounds with acoustic biosensors is easily possible at high concentrations of the target analyte, as shown by the detection of folic acid [90]. As shown in Table 2 and Table 3, competitive and binding inhibition test formats allow for the detection of lower concentrations than direct detection (Section 3.4). As a result, the direct detection of low-molecular-weight compounds meets the relevant concentration range only at high MRL values, as is the case, for instance, for the antibiotics ciprofloxacin and enrofloxacin with a sum-MRL^milk^ of 100 ng/mL [89]. Again, possible improvements regarding the LODs would include higher acoustic device frequencies and the introduction of labels. However, in order to actually compete with, for instance, the standard method of HPLC-MS, it has to be considered that the latter may allow for the detection of several residues in one sample in one measurement run [128], which would partly compensate for the increased experimental effort. Using acoustic biosensors, the detection of several analytes would require several measurement runs with single biosensors or one measurement run using a biosensor array. In both cases, several biosensor devices are required with correspondingly functionalized surfaces, depending on the analyte type and the test format. Acoustic biosensor transducers are principally compatible with array integration, particularly SAW and FBAR devices [19]. Therefore, depending on the application and the final performance, a future acoustic biosensor array might still be well suited for pre-testing or screening tasks outside a specialized laboratory.

What has been discussed so far with regard to the detection of high analyte concentrations with acoustic biosensors also applies to the detection of bacteria (Section 3.5). Direct detection of high concentrations, e.g., to determine whether probiotics contain the effective concentration of probiotic bacteria, can be performed well with acoustic biosensors (Table 4). Similar to the detection of IgG mentioned before, LFA might again be an alternative for fast screening, provided the accuracy is sufficient. Direct detection of cell counts below 100 with acoustic biosensors is possible with high-frequency devices, as shown with an EMPAS operated at 984 MHz [115], or by including separation and detection with magnetic particles (Table 5). The detection of only a few cells or only one cell, as required for pathogens, makes sample incubation and subsequent DNA amplification necessary, including the increased experimental effort (Table 6). In this context, the recently introduced lab-on-a-chip system combining LAMP with SAW sensing represents remarkable progress since it enables the detection of pathogen concentrations down to one cell in 25 mL milk in only four hours. Not only can such a small pathogen concentration be detected in such a short time, but sample processing is also simplified by having the majority of the sample-handling steps done on the chip [121,122]. This provides a powerful tool for maintaining food safety.

## 4. Conclusions

Acoustic biosensors are in the minority compared with electrochemical or optical biosensors. However, as shown by the example of milk analysis, BAW and SAW biosensors have successfully been used to detect a variety of different analytes in relevant concentrations in the complex sample matrix of milk. Applications included the determination of physical liquid parameters and the detection of a wide range of analytes, including low- and high-molecular-weight compounds and bacteria. Particularly high analyte concentrations can easily be detected with acoustic biosensors, whereas for lower concentrations and multi-analyte detection, further developments are required for acoustic biosensors to keep up with standard detection methods. Most analytical tasks were investigated using QCM biosensors, followed by SAW biosensors and EMPAS devices. Studies applying QCM biosensors were facilitated by the commercial availability of QCM sensor instruments, but specific setups were also designed. EMPAS devices are most promising regarding sensor sensitivity, but more user-friendly setups are still required. Recent developments with SAW biosensors take particular advantage of their compact design to integrate them into multifunctional devices. This latest development allows for simple and rapid detection of pathogenic bacteria in milk at the low concentrations required for food safety. Furthermore, this suggests that more analytical tasks will also be solved with acoustic biosensors in the future and, hence, investigations and further developments in the field of acoustic biosensors continue to be beneficial.

## Figures and Tables

**Table 1:** Detection of proteins (*M*_r_ > 10,000) in cow’s milk with QCM sensors.

Protein	Top Sensor Layer	LOD (Buffer)	Milk Sample: Pretreatment Steps	Achievements with Milk Samples
Immunoglobulin G (IgG)	Antibody	46 ng/mL	Raw milk: spiked, diluted (1000×)	Results achieved with 569–1675 µg/mL IgG corresponded to radial immunodiffusion (reference method) results [12]
Milk fractions	SS2343	n/a	Raw milk: untreated or processed to skim milk, whey, or permeate	Modeling milk protein adsorption (biofouling) on stainless steel in relation to milk composition and temperature at *T* = 25, 50, or 65 °C [65]
Phosphoproteins	Aminated titanium dioxide	5.3 ng/mL α-casein	Non-fat milk (protein content 3%): centrifuged, diluted	Detection of phosphoproteins in 5× to 20× diluted milk, confirmation of peptides using MALDI-ToF MS [79]
Plasmin	Cleavabl:e peptide	0.65 nM = 55 ng/mL	Milk, 2.6% fat: centrifuged, diluted, filtered, spiked	Spiking with 1, 10, or 20 nM plasmin resulted in an average recovery of 63.0% ± 0.6% [80]
	β-casein	(167.16 ± 39.36) pM = (14.2 ± 3.3) ng/mL	Milk: centrifuged, filtered, spiked	Detection of active plasmin; 70% of the β-casein layer was removed by 20 nM plasmin [81]
Staphylococcal enterotoxin A (SEA)	Antibody	20 ng/mL	Skimmed milk powder mixed with water: spiked	Spiking with 97, 194, 485, or 970 ng/mL SEA resulted in observable frequency shifts from 194 ng/mL SEA upward [82]
	Antibody	0.4 ng/mL	Milk: acidified, centrifuged, neutralized, spiked	Spiking with 5 ng/mL and 10 ng/mL SEA resulted in a recoveries of 96% and 93%, respectively [83]
SEA and staphylococcal enterotoxin B (SEB)	Corresponding MIPs	SEA: 7.97 ng/mL SEB: 2.25 ng/mL	Pasteurized milk: acidified, filtrated, neutralized, refiltered, spiked	Spiking with 5, 50, or 100 ng/mL enterotoxin resulted in recoveries ranging from 97.00% to 104.12% (SEA) and from 93.42% to 114.20% (SEB) [84]
SEB *	Antibody	2.5 µg/mL	Fresh, low fat and skimmed milk: spiked	Spiking with 0, 2.5, 5, or 10 µg/mL SEB resulted in recoveries ranging from 80% to 140% [85]

* Note: frequency responses were read out after rinsing and drying the sensor.

**Table 2:** Direct detection of low-molecular-weight compounds (*M*_r_ < 500) in cow’s milk with QCM sensors.

Low-Molecular-Weight Analyte	Top Sensor Layer, Underlying Structure	LOD (Buffer)	Milk Sample: Pretreatment Steps	Achievements with Milk Samples
Chloramphenicol	MIP, none	7 × 10^−8^ µg/mL = 7 × 10^−5^ ng/mL	Milk: diluted, filtered; standard addition method	Spiking with 1 µg/kg (≈1 ng/mL) chloramphenicol resulted in a recovery of 99.3% [86]
Ciprofloxacin, levofloxacin *	Antibody, multi-walled carbon nanotubes (MWCNTs)	Ciprofloxacin: 21 ng/mL; Levofloxacin: 25 ng/mL	Milk: diluted, mixed with organic solvent and ammonium sulfate, centrifuged; spike-and-recovery method	Spiking with 50, 100, or 300 ng/mL ciprofloxacin resulted in recoveries ranging from 98.0% to 99.0%; the same results were obtained for spiking with levofloxacin [87]
Endosulfan	MIP, microspheres	5.59 ng/mL	Pasteurized milk: spiked, acidified, filtered, neutralized, refiltered	Spiking with 5, 50, or 100 ng/mL endosulfan resulted in recoveries ranging from 101.8% to 108.0% [88]
Enrofloxacin	MIP, none	0.053 mg/L = 53 ng/mL	Pure milk: spiked, mixed with organic solvent, centrifuged, solid product extracted, supernatants filtered, dried, redissolved	LOD in milk 0.31 ng/mL; spiking with 10, 20, or 40 ng/mL enrofloxacin resulted in recoveries ranging from 77.2% to 84.2% [89]
Folic acid	MIP with metal chelate, none	0.0080 μM = 3.5 ng/mL	Follow-on baby milk with a defined folic acid content: diluted using an organic solvent/water mixture	Folic acid content was 150 ng/mL and 180 ng/mL; recoveries obtained with QCM (HPLC-UV/VIS) were 91.9% and 94.0% (103.1% and 103.0%), respectively [90]
Melamine	MIP, none	8 µM = 1 µg/mL	Skimmed milk (0.5% fat, 3.5% protein) and natural whey (0.1% fat, 0.6% protein): spiked, partly diluted or centrifuged	Observable frequency shifts after spiking with 3200 µg/mL melamine only in whey, in skimmed milk only after 10× dilution, or if the spiking was done after centrifugation; i.e., direct melamine detection is hindered by the interaction between melamine and protein [91]
	MIP, none	1.8 × 10^−8^ M = 2.3 ng/mL	Pasteurized milk: acidified, supernatant spiked and neutralized, mixed with an organic solvent, centrifuged, diluted	Higher frequency shifts with milk samples spiked with melamine up to 1000 ng/mL compared with non-spiked samples only if spiking was done after milk protein precipitation, avoiding joint precipitation of the proteins with melamine [92]
Methimazole	MIP, hollow spheres	3 ng/mL	Milk: spiked, incubated overnight, mixed with an organic solvent, centrifuged, extracted, dried, redissolved	Spiking with 50, 100, or 200 ng/mL methimazole resulted in recoveries ranging from 89.57% to 101.97% [93]
Tobramycin	MIP, none	5.7 pM = 0.0027 ng/mL	Milk: acidified, centrifuged, precipitate extracted, supernatants centrifuged and filtered	Spiking with 10, 40, or 60 pM = 0.0047, 0.0187, or 0.0281 ng/mL tobramycin resulted in recoveries ranging from 97% to 98% [94]

* Note: frequency responses were read out after rinsing and drying the sensor.

**Table 3:** Detection of low-molecular-weight compounds (*M*_r_ < 500) in cow’s milk using competitive and binding inhibition assays with QCM and SAW sensors.

Sensor Device	Assay Format	Low-Molecular-Weight Analyte	Top Sensor Layer	LOD (Buffer)	Milk Sample: Pretreatment Steps	Achievements with Milk Samples
QCM	Competitive	Ampicillin, penicillin G	Hapten–protein conjugate	Ampicillin 3.9 ng/mL; penicillin G 0.8 ng/mL	Milk: mixed with ammonium sulfate, centrifuged; added-found method	Spiking with 10 ng/mL or 20 ng/mL ampicillin and/or penicillin G led to recoveries of 126% and higher [95]
		Chloramphenicol	Hapten–protein conjugate	0.2 ng/mL	Milk: mixed with ammonium sulfate, centrifuged; added-found method	Spiking with 5 ng/mL and 10 ng/mL chloramphenicol resulted in recoveries of 80% and 90%, respectively [96]
		Ciprofloxacin, levofloxacin *	Hapten–protein conjugate (via MWCNTs)	Ciprofloxacin: 8 ng/mL; levofloxacin: 9 ng/mL	Milk: diluted, mixed with organic solvent and ammonium sulfate, centrifuged; spike-and-recovery method	Spiking with 20, 50, or 70 ng/mL ciprofloxacin resulted in recoveries ranging from 95.5% to 103.2%; for spiking with the same concentrations of levofloxacin recoveries were 97.5% to 103.6% [87]
		Zearalenone	Hapten–protein conjugate	0.37 ng/mL	Milk: spiked, mixed with a diluted organic solvent, centrifuged, supernatant dried, redissolved	Spiking with 5, 50, or 100 ng/mL zearalenone resulted in recoveries ranging from 78.8% to 89.0%; comparable to HPLC-MS/MS with recoveries ranging from 80.1% to 90.5% [97]
	Competitive with gold-labeled antibodies	Diethyl-stilbestrol *	Hapten–protein conjugate	13 ng/mL	Milk: spiked, mixed with organic solvent, collection and dilution of supernatant	Spiking with 0.5, 5, or 50 ng/mL diethylstilbestrol resulted in recoveries ranging from 98.0% to 104.8%; comparable to HPLC-MS/MS with recoveries ranging from 102.0% to 104.9% [98]
	Indirect competitive	Aflatoxin B1	Hapten–protein conjugate	0.01 ng/mL	Whole fat milk, light milk or skim milk powder dissolved in water: spiked, mixed with an organic solvent, centrifuged, diluted	Spiking with 0.1 ng/mL and 10 ng/mL aflatoxin B1 and using gold-labeled secondary antibodies resulted in recoveries ranging from 95.0% to 107% [99]
				0.01 ng/mL	Whole fat milk, light milk or skim milk powder dissolved in water: centrifuged, mixed with an organic solvent, filtered, diluted	Spiking with 0.1, 1, or 10 ng/mL aflatoxin B1 and using horseradish peroxidase (HRP)-labeled secondary antibodies for the biocatalyzed precipitation of an insoluble product resulted in recoveries ranging from 94.6% to 110% [100]
SAW resonator	Binding inhibition	Penicillin G	Hapten–hydrogel	n/a	Low-fat milk (1.3% fat): untreated or centrifuged, spiked	Samples spiked with 0, 2, 4, 6, 8, or 10 ng/mL penicillin G could be classified to be below or above 4 ng/mL (maximum residue limit (MRL)) [13]

* Note: frequency responses were read out after rinsing and drying the sensor.

**Table 4:** Detection of probiotic and pathogenic bacteria in cow’s milk with QCM sensors and EMPAS devices without the use of sample enrichment methods other than spiking or incubation.

Sensor Device	Assay Format	Bacterium	Top Sensor Layer	LOD (Buffer)	Milk Sample: Pretreatment Steps	Achievements with Milk Samples
QCM	Direct detection	*Bifidobacterium bifidum*, *Lactobacillus acidophilus*	Corresponding antibody	10^4^ CFU/mL for each	Low-fat UHT milk (1.5% fat): spiked, some of them fermented (i.e., incubated) up to 24 h, all filtrated and diluted	Similar results for *B. bifidum* and *L. acidophilus* in 100× diluted spiked and fermented milk samples: LOD 10^3^ CFU/mL; measuring range 10^3^ CFU/mL to 5 × 10^5^ CFU/mL, the cell numbers obtained via QCM measurements correlated with those from plate count [106]
		*Escherichia coli*	Gold (QCM electrode)	1.1 × 10^7^ CFU/mL	Milk: spiked, incubated (5 h), centrifuged, cell pellet resuspended	Sample concentrations determined with QCM sensors were in the range of 9.18 × 10^7^ CFU/mL to 1.93 × 10^8^ CFU/mL, which was comparable to the results obtained from a plate count [108]
			Parylene C	10^2^ cells/mL	Milk: spiked, incubated (3 h)	Time-resolved monitoring of cell population growth [109]
			Antibody	1.7 × 10^5^ CFU/mL	Milk: untreated or diluted, spiked or inoculated with 10^2^ CFU/mL *E. coli* and incubated (18 h)	Spiked and incubated milk samples containing 10^7^ CFU/mL and 10^6^ CFU/mL *E. coli* led to frequency shifts of 88.0 Hz ± 23.6 Hz and 52.0 Hz ± 23.1 Hz, respectively, which were higher than those obtained with untreated milk, i.e., 21.1 Hz ± 13.5 Hz [110]
		*Francisella tularensis*	Antibody	10^5^ CFU/mL	Low-fat UHT milk (1.5% fat): spiked	LOD in milk 10^5^ CFU/mL; spiking with 10^5^ CFU/mL (10^8^ CFU/mL) *F. tularensis* resulted in significantly higher signals than spiking with the same concentrations of *E. coli* and *Bacillus subtilis* [111]
		*Salmonella enterica*	Antibody	n/a	Milk: spiked	Frequency shifts obtained by spiking with 1.2 × 10^7^ CFU/mL to 4.8 × 10^7^ CFU/mL *S. enterica* fitted within the calibration range obtained with 3.2 × 10^6^ CFU/mL to 4.8 × 10^8^ CFU/mL in a culture broth [112]
QCM	Displacement	*Listeria monocytogenes*	Cell-antibody complex	n/a	Milk (2% fat): spiked	Spiking with 3.19 × 10^6^ and 6.38 × 10^6^ *Listeria* cells resulted in significantly higher slopes than spiking with 6 × 10^6^ cells of non-specific *Serratia* [113]
QCM	Sandwich, gold-labeled 2nd antibody	*B. bifidum*	Antibody	2.1 × 10^2^ CFU/mL	Fresh milk: diluted (1 g milk in 50 mL buffer), spiked, incubated (2 h)	Spiking with 10^4^ CFU/mL of *B. bifidum* resulted in significantly higher signals than with non-specific *L. acidophilus*, *L. monocytogenes*, and *E. coli* [114]
EMPAS	Direct detection	*E. coli*	Aptamer	35 CFU/mL	UHT milk (3.5% fat): spiked	LOD in milk 8 CFU/mL; recovery of 127.4% in spiked milk samples [115]

**Table 5:** Direct detection of pathogenic bacterial cells in cow’s milk with QCM sensors, after being separated with correspondingly coated magnetic particles.

Bacterium	Top Sensor Layer	LOD (Buffer)	Milk Sample: Pretreatment Steps	Achievements with Milk Samples
*Brucella melitensis*	Aptamer	10^2^ CFU/mL	Milk: spiked, mixed with aptamer-coated magnetic particles, magnetic separation, washing and elution of captured bacteria	LOD in milk 10^3^ CFU/mL; spiking with 11,780 cells of *B. melitensis* was evaluated as 10,052 cells with QCM sensor, while 9827 cells of non-specific bacteria of the same genus (*B. suis*) resulted in sensor signals below sensitivity [116]
*E. coli* *	Antibody	23 CFU/mL	Milk: spiked, mixed with antibody-coated magnetic particles (anti-*E. coli* + biotin antibody), magnetic separation and washing; mixing with streptavidin–gold, magnetic separation and washing, catalytic growth of the gold, magnetic separation, washing, and resuspension of pellet consisting of particles, gold, and captured cells	LOD in milk 53 CFU/mL [117]
*L. monocytogenes*	Antibody	3 cells per 200 µL sample, i.e., 15 cells/mL	UHT sterile milk (0.1% fat): spiked, mixed with antibody-coated magnetic particles, separation, washing and resuspension of pellet consisting of particles and captured cells	LOD in milk was 3 cells per 200 µL sample, i.e., 15 cells/mL; if magnetic particle enrichment was omitted, no frequency shift was obtained for milk spiked with *Listeria* up to 10^8^ CFU/mL [118]
*S. enterica*	Aptamer	10^2^ CFU/mL	Milk (≥ 1.5% fat): spiked, mixed with aptamer-coated magnetic particles, magnetic separation, washing and elution of captured bacteria	Spiking with 10^4^ CFU/mL *Salmonella* resulted in significantly higher signals than obtained with 10^4^ CFU/mL non-specific *Escherichia*, but only if magnetic particle enrichment was applied [119]

* Note: frequency responses were read out after rinsing and drying the sensor.

**Table 6:** Detection of probiotic and pathogenic bacteria in cow’s milk using cell lysis and DNA-amplification tools.

Sensor Device	Bacterium	Top Sensor Layer	LOD (Buffer)	Milk Sample: Pretreatment Steps	Achievements with Milk Samples
QCM	*E. coli*	Oligonucleotide	Direct detection: 1.2 × 10^4^ CFU/mL; sandwich assay with gold-labeled oligonucleotides: 1.2 × 10^2^ CFU/mL	Pasteurized milk: spiked, mixed with proteinase K and Triton X-100 for 1 h, mixed with NaCl, centrifuged, collection and purification of cell pellet, passing pellet on to genomic DNA extraction and PCR	Spiking milk with 5.3 × 10^2^ CFU/mL *E. coli* and application of sandwich hybridization with gold-labeled oligonucleotides resulted in significantly larger frequency shifts than obtained with non-spiked milk [120]
SAW delay line	*S. enterica*	Poly(L-lysine)	See “Achievements with milk samples”	Whole UHT milk (3.5% fat): spiked, mixed with antibody-coated magnetic particles, incubated (3 h), magnetic separation of pellet consisting of particles and captured bacteria, resuspension (buffer), lysis, LAMP	LOD in milk ~3 aM DNA target or 2 cells/µL; processing of 25 mL milk spiked with 1–25 CFU of *S. enterica* reveals a minimum detectable content of 1 cell in 25 mL milk [121]
	*S. enterica*, *E. coli*, *Bacillus cereus*, *Listeria*	Poly(L-lysine)	See “Achievements with milk samples”	fresh milk (full fat): spiked, incubated (3 h), centrifuged, resuspension of bacteria pellet (buffer) and injection in a chip for bacteria to be captured using an antibody-coated zone, lysis, LAMP	Processing of 25 mL milk spiked with 1–5 CFU of bacteria allows for the detection of 1–5 cells in 25 mL milk [122]

## Data Availability

Details on the literature search conducted for this review paper, including the search terms and selection parameters, are available from the corresponding author on request.
